# The British Nurses' Association

**Published:** 1888-07-21

**Authors:** H.R.H. Princess Christian

**Affiliations:** President


### The British Nurses' Association.

By H.R.H. Princess Christian (President) and Mr. Burdett.

Being Letters reprinted from *The Times* of the 11th and 18th inst.

As president of the British Nurses' Association, a society which is designed "to unite all British nurses for their mutual help and protection, and for the advancement in every way of their professional work," I am desirous to place before the public, through your columns, a short statement of some of the methods by which the objects of the association will, we hope, be attained, of the advantages which will accrue from their attainment, and of the methods by which such attainment may be promoted by those who are not included within our body.

It is hardly necessary to say that the education of trained nurses in this country has been greatly due to the experience of the Crimean War, and to the noble efforts then made by Miss Nightingale to provide for the proper care of the sick and wounded soldiers. At the present day trained nurses are said to amount to 15,000 in number; and, whether in hospitals or in private families, their aid in sickness is universally held to be indispensable. No surgeon or physician would willingly take charge of a serious case without the assistance of a trained nurse, and the medical profession as a body has borne the most prompt and generous testimony to the great value of skilled nursing. There must have been countless instances, there must be countless English homes, in which the presence of a trained nurse, knowing what to do and to avoid, and capable of rendering intelligent obedience to medical directions, has turned the scale between life and death.

Nurses are trained in hospitals, and, in order that their training may be effectual and may fit them for all the duties which they may be called upon to discharge, it must be of considerable duration, must embrace a considerable variety of experience, and must be guided by careful and systematic teaching. A nurse must attend lectures, and must pass through medical, surgical, and special wards before she can be fully capable of doing all which can be required of her. In other words, her professional education can only be completed at the cost of much time, industry, and expense. Student nurses, or "probationers," in many cases pay to be taught, and, even when they do not actually give money, they are required to work for a smaller remuneration than they might easily obtain in other kinds of employment.

The natural goal of any distinctly professional education is the attainment of some title which may be accepted by the public as an evidence of competency, but in the case of nurses, although the education has now been given for several years, the natural goal has still to be supplied. Many women who enter as probationers are found to be physically or morally unfit for the duties of nursing, and are dismissed from training by their superiors, while many others withdraw themselves after a brief period of instruction. Members of both these classes, although not only incompetent but possibly otherwise unfit, are apt to seek employment in nursing and to describe themselves as "hospital trained nurses."

Against such unqualified persons it is in the power of hospitals to protect themselves, but it is hardly in the power of the public to do the same. Many private agencies for the supply of nurses to families have come into existence, and the number of such agencies seems likely to increase. Although there can be no doubt that many of those which now exist are admirably conducted, and are of great public utility, there is, nevertheless, reason to fear that the managers of others do not always require sufficient security for the fitness of those whom they engage. A nurse, when wanted, is generally wanted at once, and a private employer is seldom able to test her qualifications. He is almost compelled to trust to some agency or institution, and is, to this extent, liable to be deceived.

The British Nurses' Association, having succeeded, in the first place, in constituting itself by the enrolment as members of thoroughly trained and competent nurses and no others, is now seeking to establish, it is hoped on the secure basis of a Royal charter, a system of registration which will enable every trained nurse to produce documentary evidence of her education and attainments, and thus to show that she is entitled to confidence in her calling. When such a system is brought into operation, people who employ unqualified nurses will have only themselves to blame for any ill-consequences which may ensue.

Besides this, the primary object of the association, it has other objects also, which are, indeed, subsidiary to the first, but which will scarcely be less useful. It aims at affording increased facilities for the education of nurses, and it will also seek to originate and to maintain, when necessary, by the aid of the public, whatever institutions or helps may seem likely to be beneficial to nurses generally, and calculated to improve the quality of their work. It is hardly within the power of nurses, by the small annual contributions which are all that they can afford, to bear the whole burden of supporting the association in order to carry out even its primary object, and it is still less within their power to meet the cost which the attainment of the secondary objects must entail. These, while they are important to nurses themselves, are in at least an equal degree important to the public, who not only constantly require the aid which nurses can give, but who would derive very real and practical advantages from being rendered able to protect themselves against the pretensions of unskilled persons. It is especially on this latter ground that I am desirous to ask for pecuniary help towards the complete realisation of our plans. It is well known with what bountiful liberality four great mercantile firms have endowed a fund for the benefit of nurses who are past work, and I am hopeful that some similar response will be made to an appeal which has for its object to increase the efficiency of those who are still at the post of duty.

Contributions for this purpose will be thankfully received, and may be paid either to the bankers of the association, Messrs. Barclay, Bevan, Tritton, Ransom, Bouverie, and Co., 54, Lombard Street, E.C.; or to the honorary secretaries, Dr. Bedford Fenwick and Miss C. J. Wood, at 20, Upper Wimpole Street, W.

---

By Mr. Henry C. Burdett.

The letter of H.R.H. Princess Christian in the *Times* of the 11th inst. has caused surprise in many quarters. It has, heretofore, been stated on authority, that the chief object of the British Nurses' Association was to raise the status of the whole body of nurses by organising its members, and so elevating nurses into a distinct profession. It could, therefore, no more appeal for eleemosynary aid than the British Medical Association, for example. Indeed, so far has this spirit of independence been carried, that those who sympathise with it have denounced the eleemosynary element in the National Pension Fund for Nurses as "insulting;"

they have referred to it as "the professed dole," which is "not only superfluous," but to be resented "as an offer of undemanded charity." The grounds of this condemnation of the introduction of the eleemosynary element in the Pension Fund, which is instituted for the benefit of all nurses without distinction, is, that the highest class of nurses, to whom nursing is a profession —*i.e.*, all the members of the British Nurses' Association —say that the proper objects of a compassionate fund are such as are the victims of misfortune, not such as are capable of the efficient pursuit of a chosen profession. The representatives of the chief metropolitan hospitals met at Guy's Hospital a few days ago to consider the means of the nurses employed by them, and they were unanimously of opinion that the views of the promoters of the National Pension Fund were correct, as they knew their nurses could not, without eleemosynary aid, provide an adequate annuity out of their limited earnings. The appeal now issued by the Princess Christian for charitable contributions to the British Nurses' Association would seem to be an admission on the part of that Association that the views they originally held on this point must be modified if their work is to be brought to a successful issue. Is this really so?

Again, although nursing institutions and hospital training schools are mainly supported by, and under the management of laymen, *i.e.*, members of the general public who subscribe to such institutions, the founders of the British Nurses' Association "for the attainment of its objects, and in order that it may be so conducted as to promote the best interests of its members, have felt it necessary that it should be strictly limited to nurses and to medical practitioners, to the exclusion of all other persons." This was all very well so long as contributions were not invited from "all other persons," but now a new departure has taken place, and, if I may say so without offence, more practical counsels have prevailed, must not some place in the constitution be found for those who provide the sinews of war? It has always been admitted from time immemorial that he who pays the piper has the right to select the tune, and so the managers of hospitals and nursing institutions must now necessarily be included in the constitution of the British Nurses' Association.

If both these points are conceded, as they are believed to be conceded by Princess Christian's letter, might I suggest that the time has arrived for summoning a conference of those who are interested in the welfare of nurses or in the management of institutions, hospitals, pension funds, or other schemes for their benefit, so that all overlapping and wastefulness may be prevented, and the utmost value may be obtained for the money which the public may subscribe for the benefit of all classes of nurses.

